# Assessment of Aptamer as a Potential Drug Targeted Delivery for Retinal Angiogenesis Inhibition

**DOI:** 10.3390/ph16050751

**Published:** 2023-05-16

**Authors:** David Moreira, Jéssica Lopes-Nunes, Fátima Milhano Santos, Maria Paula Cabral Campello, Maria Cristina Oliveira, António Paulo, Cândida Tomaz, Carla Cruz

**Affiliations:** 1 CICS-UBI—Health Sciences Research Centre, University of Beira Interior, 6201-506 Covilhã, Portugal; 2 Functional Proteomics Laboratory, Centro Nacional de Biotecnología, Consejo Superior de Investigaciones Científicas (CSIC), Calle Darwin 3, Campus de Cantoblanco, 28049 Madrid, Spain; 3 Centro de Ciências e Tecnologias Nucleares, Instituto Superior Técnico, Universidade de Lisboa, Estrada Nacional 10 (km 139.7), 2695-066 Bobadela, Portugal; 4 Departamento de Engenharia e Ciências Nucleares, Instituto Superior Técnico, Universidade de Lisboa, Estrada Nacional 10 (km 139.7), 2695-066 Bobadela, Portugal; 5 Departamento de Química, Universityof Beira Interior, Rua Marquês de Ávila e Bolama, 6201-001 Covilhã, Portugal

**Keywords:** G-quadruplex aptamers, retinal diseases, nanosystems, nucleolin, angiogenesis

## Abstract

AT11-L0 is an aptamer derivative of AS1411 composed of G-rich sequences that can adopt a G-quadruplex (G4) structure and target nucleolin (NCL), a protein that acts as a co-receptor for several growth factors. Hence, this study aimed to characterize the AT11-L0 G4 structure and its interaction with several ligands for NCL targeting and to evaluate their capacity to inhibit angiogenesis using an in vitro model. The AT11-L0 aptamer was then used to functionalize drug-associated liposomes to increase the bioavailability of the aptamer-based drug in the formulation. Biophysical studies, such as nuclear magnetic resonance, circular dichroism, and fluorescence titrations, were performed to characterize the liposomes functionalized with the AT11-L0 aptamer. Finally, these liposome formulations with the encapsulated drugs were tested on the human umbilical vein endothelial cell (HUVEC) model to assess their antiangiogenic capacity. The results showed that the AT11-L0 aptamer–ligand complexes are highly stable, presenting melting temperatures from 45 °C to 60 °C, allowing for efficient targeting of NCL with a *K*_D_ in the order of nM. The aptamer-functionalized liposomes loaded with ligands C_8_ and dexamethasone did not show cytotoxic effects in HUVEC cells compared with the free ligands and AT11-L0, as assessed by cell viability assays. AT11-L0 aptamer-functionalized liposomes encapsulating C_8_ and dexamethasone did not present a significant reduction in the angiogenic process when compared with the free ligands. In addition, AT11-L0 did not show anti-angiogenic effects at the concentrations tested. However, C_8_ shows potential as an angiogenesis inhibitor, which should be further developed and optimized in future experiments.

## 1. Introduction

Pathological angiogenesis in the eye may be caused by the deregulation of retinal homeostasis, which belongs to a group of diseases known as neovascular retinal diseases [1,2]. Some of the principal diseases that lead to this pathological angiogenesis are diabetic retinopathy (DR), age-related macular degeneration (AMD), retinopathy of prematurity (ROP), and retinal arterial or vein occlusion [3,4,5]. The angiogenic process combined with the disruption of the blood–retinal barrier (BRB) in these diseases can lead to irreversible blindness [6]. Usually, retinal angiogenesis is triggered by a hypoxia episode but it may be caused by other pathological processes such as inflammation, neurodegeneration, vascular damage, and oxidative stress [4,7,8,9,10]. The principal molecule involved in this process is vascular endothelial growth factor (VEGF), but other cytokines such as platelet-derived growth factor (PDGF), transforming growth factor beta (TGF-β), interleukin-1 (IL-1), interleukin-6 (IL-6), and complement molecules (e.g., C5 and C3) also play relevant roles [11,12]. Regarding this overexpression of inflammatory cytokines, the use of monoclonal antibodies against VEGF has been established as a valuable alternative to more traditional treatments (surgery and laser photocoagulation) [11,13]. The most used anti-VEGF antibodies are ranibizumab, bevacizumab, aflibercept, and faricimab [14,15,16]. However, the therapeutic use of these antibodies presents some drawbacks such as their high cost, short half-life, the occurrence of side effects (e.g., inflammation, retinal detachment, or increased ocular pressure), or the need for repeated intravitreal injections to administrate the antibodies. Furthermore, some patients do not respond favorably to anti-VEGF, and it is recommended to switch the treatment to corticosteroids such as dexamethasone [17]. As an alternative to antibodies, aptamers have emerged as a promising approach to inhibit the angiogenic process of the retina [18,19,20].

Aptamers are small nucleic acid sequences that interact with one specific target just as happens with antibodies. However, compared to antibodies, aptamers have several advantages including their lower cost, lower immunogenicity, and increased half-life, and they are also easier to synthesize and modify [18,19,20]. Regarding these advantages, aptamers have been used to target VEGF, similarly to antibodies. The first approved aptamer for VEGF targeting was Macugen^®^ [21]. Despite all the potential advantages of aptamers, Macugen^®^ only targets one specific isoform of VEGF (VEGF-165), and its therapeutic efficacy is lower than expected. Other aptamers have already entered clinical trials, such as Fovista^®^ and Zimura^®^, which target PDGF and C5 molecules, respectively [22,23,24]. Another important protein that may have a role in these diseases is nucleolin (NCL), a ubiquitous protein with important regulatory roles in the nucleus, which acts as a co-receptor of growth factor [25]. As a result of VEGF overexpression, NCL is phosphorylated and translocated into the cell membrane where it acts as a co-receptor for several growth factors [26]. Indeed, several studies have highlighted the important role of NCL in angiogenic in neovascular retinal diseases, since its inhibition promotes an antiangiogenic effect [27].

AS1411 is another aptamer that is being greatly studied for NCL targeting [28,29]. One of the most interesting aspects of this aptamer is that it adopts a G-quadruplex (G4) structure whose stability depends on the salt concentration in the environment. The tri-dimensional structure of aptamers is the basis for the specific interaction with their target, and the G4 structure is important for NCL targeting [30,31]. The AS1411 aptamer has already been studied for the inhibition of NCL on retinal angiogenesis [28,29]. However, due to its high polymorphism, it is not known which conformation is the most biologically relevant. To overcome this problem, AS1411 derivatives have been developed, namely, AT11. AT11 and its derivatives have small variations in the bulge/linker size from zero to two thymines [32]. Hence in this study, we evaluated the G4 stability of AT11-L0 formulated as a liposome containing anti-inflammatory dexamethasone or an acridine orange derivative termed C_8_ to reduce pathological angiogenesis.

## 2. Results

### CD Spectroscopy Measurements

**Figure 1:**
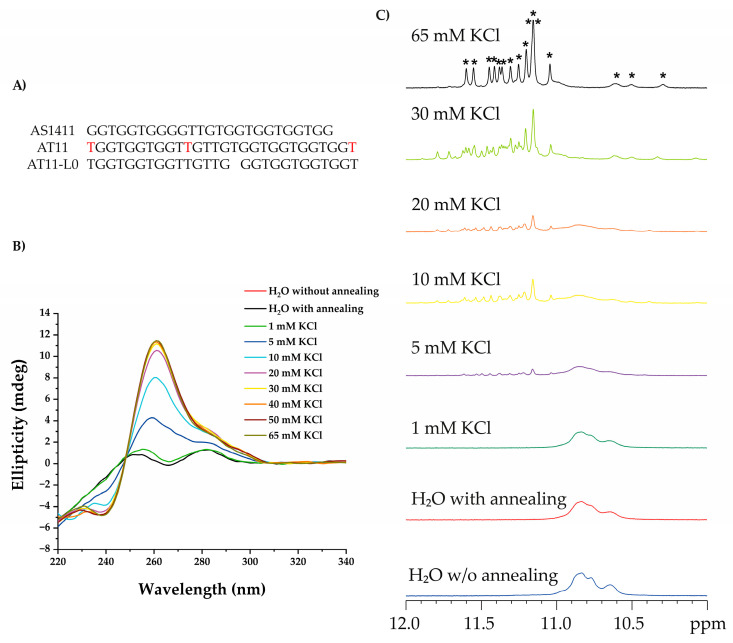
Evaluation of G4 formation using distinct spectroscopic techniques. (**A**) AS1411 derivatives. (**B**) CD spectra of AT11-L0 (10 μΜ) obtained with increasing concentrations of KCl in 20 mM of phosphate buffer in the range of 220–340 nm. (**C**) The effect of KCl salt on the structure formation of AT11-L0 G4 (50 μΜ) was monitored by ^1^H NMR spectroscopy recorded in potassium buffer supplemented with 10% D_2_O and increased concentrations of KCl (0 to 65 mM) (all regions of spectra in Figure S1). Imino protons of AT11-L0 are indicated with asterisks (∗). All measurements were performed at 20 °C.

Aptamers that contain G-rich sequences adopt a G4 conformation due to internal and/or inter-strand folding by hydrogen bonds. This is the case for aptamer AS1411 and its derivative AT11 (Figure 1A), which form a single major G4 conformation but exhibit anti-proliferative activity similar to that of AS1411 [33]. Modifications on linker/bulge/loop elements of AT11 G4 were achieved, resulting in AT11-L0, which has no linker.

Firstly, we evaluated the ability of the AT11-L0 to form a G4 structure under different concentrations of KCl using CD spectroscopy. The obtained spectra (Figure 1B) showed that AT11-L0 can fold into a stable parallel G4 at 10 mM KCl with the typical positive band at 260 nm and the negative one at 240 nm. The stabilization of the parallel G4 was achieved with an increase in the ellipticity at 260 nm and a decrease at 240 nm at 30 mM KCl. The KCl concentration-dependent stabilization remained constant between 30 mM and 65 mM. Regarding that, a well-defined G4 parallel structure was observed at concentrations above 30 mM of KCl.

To confirm these results, ^1^H NMR titrations were performed at different concentrations of KCl. The results also indicated that G4 was formed above 30 mM of KCl. The NMR spectra (Figure 1C) showed 16 peaks between 10 and 12 ppm resulting from the 16 guanines that form the Hoogsteen hydrogen bonds. These 16 imino peaks associated with guanines in the G-tetrad formation were compatible with a G4 structure formed by two subunits of two G-tetrads each, as previously reported [33,34].

**Figure 2:**
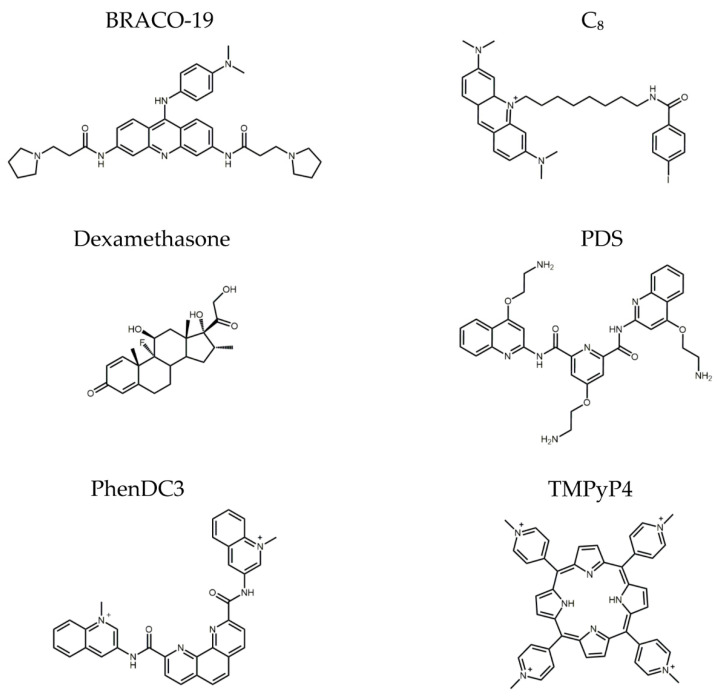
Chemical structure of molecules tested: BRACO-19, C_8_, dexamethasone, PDS, PhenDC3, and TMPyP4.

The ability of BRACO-19, C_8_, dexamethasone, PDS, PhenDC3, and TMPyP4 (Figure 2) to bind and stabilize the G4 in AT11-L0 was then evaluated through FRET melting, CD melting, and fluorescence titrations. These experiments were carried out to understand if these ligands can stabilize the AT11-L0 structure and improve its interaction with NCL. In addition, we wanted to confirm if it was possible to use the G4 structure of AT11-L0 as a delivery system of dexamethasone, an anti-inflammatory drug used for the treatment of ocular diseases [35].

**Figure 3:**
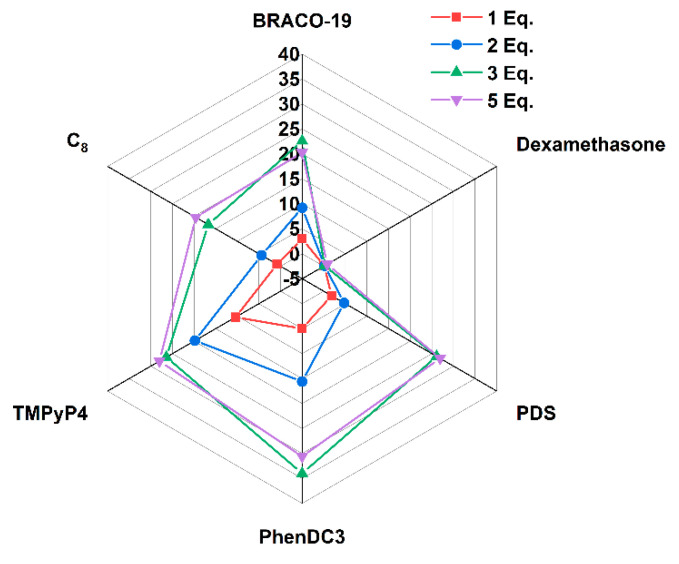
*T*_m_ radar plot of AT11-L0 in the presence of different molar equivalents of ligands obtained by FRET-melting experiments.

The FRET-melting assay is based on the stabilization induced by the ligand, leading to a difference in melting temperature between the nucleic acid alone and in the presence of this ligand. The results are presented in Figure 3. The melting temperature (*T*_m_) of AT11-L0 was 48.1 °C when pre-annealed at a 20 mM phosphate buffer (pH 6.9) containing 65 mM KCl. This result is similar to the one described by Phan et al., who reported a *T*_m_ of 51 °C [33]. The slight difference in the *T*_m_ obtained can result from the presence of the FAM and TAMRA probes. For all the ligands tested, the thermal stability of the structure increased in a concentration-dependent manner. Dexamethasone, even at 5 molar equivalents, displayed the lowest stabilization of the G4 in AT11-L0. However, at 5 molar equivalents, PhenDC3 was a strong stabilizer of G4 with Δ*T*_m_ > 30 °C. BRACO-19, PDS, and TMPyP4 also presented strong stabilization value with Δ*T*_m_ of 20.3, 26.9, and 28 °C, respectively. C_8_ also had a stabilization effect, with a Δ*T*_m_ of 19.6 °C.

At 1 molar equivalent, the ligand that promoted the highest Δ*T*_m_ was TMPyP4, with a *T*_m_ of 10.4 °C. PhenDC3 and BRACO-19 also presented evident stabilization of the G4 structure with Δ*T*_m_ values of 5 and 3 °C, respectively. At 1 molar equivalent, C_8_ and dexamethasone did not promote considerable stabilization of the structure, showing Δ*T*_m_ values of 0.8 and 0.1 °C, respectively.

A competitive FRET assay was also performed to understand if the ligand binds preferentially to the AT11-L0 G4 structure in the presence of a double-strand competitor (ds26). To quantify the selectivity of tested ligands, the S factor was calculated as a ratio of the thermal stabilization promoted by the ligand before and after the presence of the double-strand competitor. Results are highlighted in Table 1. Ligands with S factors near 1 were more selective to the AT11-L0 structure, and thons with S factors near 0 were the less selective ones. Thermal stabilization induced by PhenDC3 was not strongly affected by the ds26 competitor, since the S value was 0.89 at 50 molar equivalents. The presence of the competitor strongly affected the TMPyP4, C_8_, and BRACO-19 thermal stabilization of AT11-L0 G4, since they presented low S values (0.33, 0.38, and 0.45, respectively, at 50 molar equivalents). PDS thermal stabilization was also affected by the presence of the competitor, as its S factor was 0.73 at 50 molar equivalents. Dexamethasone was not included in this assay due to its low thermal stabilization on the AT11-L0 G4 structure. These results indicated that PhenDC3 is the most selective ligand for the AT11-L0 G4 structure.

Based on the results obtained by the FRET-melting assay, PhenDC3, C_8_, and dexamethasone were chosen for further evaluation by CD melting. Despite its low stability for G4 in AT11-L0, dexamethasone was included due to its therapeutic effect as an anti-inflammatory drug.

Firstly, the *T*_m_ of AT11-L0 G4 was determined in the absence of ligands and then in the presence of the three selected ligands at the maximum of CD ellipticity (260 nm). A lower *T*_m_ would lead to incomplete folding at low temperatures, whereas a higher *T*_m_ would prevent the analysis of the transitions obtained with highly stabilizing ligands.

*T*_m_ corresponds to the temperature at which half (*T*_1/2_) of the G4 is unfolded, and *T*_1/2_ is defined as the temperature for which the normalized ellipticity is 0.5. The *T*_1/2_ obtained for AT11-L0 G4 at 100 mM KCl was 49.3 °C, meaning that is the G4 stabilization at this KCl concentration.

After ligand addition, an increase in *T*_1/2_ is observable or not depending on the stabilization induced by the G4 ligand. In the case of adding 2 molar equivalents of dexamethasone to AT11-L0 G4, the resulting melting curve almost overlapped the AT11-L0 curve without the ligand, indicating that dexamethasone did not lead to a stabilization of the G4 in AT11-L0. The *T*_1/2_ for AT11-L0 G4 with dexamethasone was 1.71 °C. However, after the addition of 2 molar equivalents of C_8_ and PhenDC3, the melting curves deviated to higher temperatures, with a *T*_1/2_ of approximately 6.68 °C, indicating ligand-induced stabilization of the AT11-L0 G4 structure. The *T*_m_ values obtained by CD melting are presented in Table S1 and the curves in Figure S2.

Interestingly, the CD-melting experiments showed greater stabilization of unlabeled AT11-L0 oligonucleotide than did the FRET-melting experiments. To clarify this issue, CD spectra of the two AT11-L0 sequences (labeled and unlabeled) were recorded in 20 mM KPi + 65 mM KCl, and both adopted parallel G4 (Figure S3). This implies that the dyes had a minimal influence on the folding of this type of G4. However, there were small changes in the intensity and deviation of the positive peak at 260 nm and the intensity of the negative one at 240 nm; in addition, the *T*_m_ was higher for the dye-modified oligonucleotide (Figure S3), confirming the reported interpretation that the equilibrium was shifted towards the G4 structure in the presence of the dyes [36].

These results had already been obtained for promoter sequence c-myc [37], and two factors may explain this apparent discrepancy: (i) in FRET melting, we used a dual-labeled AT11-L0 sequence, while in CD we used unlabeled AT11-L0 oligonucleotide, and the two probes (FAM and TAMRA) on a G4-forming sequence can affect its stability and interaction with ligands. Indeed, the ligands can interact with the fluorescent probes, which may cause variations in the normal emission of fluorescence; (ii) oligonucleotide and ligand concentrations are different, due to the different sensibilities of the two techniques [37].

The dissociation constants (*K*_D_) of these ligands were further determined by fluorometric titrations using the Cy5-AT11-L0. Upon the addition of each ligand to the pre-annealed labeled aptamer, the fluorescence decreased due to the interaction between the aptamer and the ligand. The titration data were fitted using the most suitable method for each ligand. Dexamethasone and C_8_ were fitted with the Hill saturation binding model and PhenDC3 with the two-site bind model (Figure S4). For the tested ligands, the *K*_D_ was in the order of nM. According to the Hill model, dexamethasone and C_8_ have *K*_D_ values of 4.54 × 10^−9^ M and 22.1 × 10^−9^ M, respectively. These values indicate that both molecules have a high affinity to AT11-L0 G4 despite dexamethasone not showing thermal stabilization towards the G4 structure. In the case of PhenDC3, the two-site binding model showed that it interacted with the aptamer with high affinity (*K*_D_ = 0.329 × 10^−9^ M) at the beginning. However, with increasing concentrations of PhenDC3, the affinity became lower (*K*_D_ = 0.011 M), indicating that the preferential interaction site between the aptamer G4 and PhenDC3 may saturate at a low concentration, and then they may interact at another site but with a low affinity.

**Figure 4:**
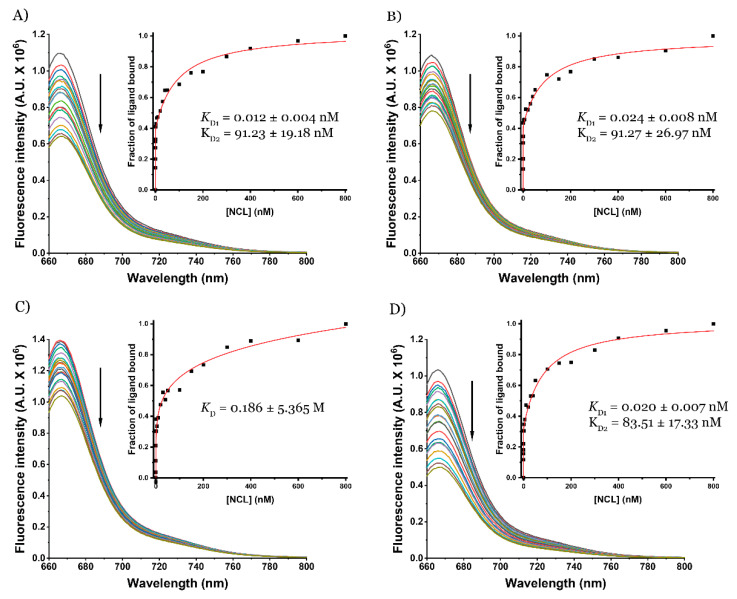
Fluorescence titrations of pre-folded (**A**) 5′-Cy5-AT11-L0 sequence, complexed with (**B**) C_8_, (**C**) dexamethasone, and (**D**) PhenDC3 with increasing concentrations of NCL.

AT11-L0 is a derivative of AT11, which itself is a derivative of AS1411, a known NCL aptamer. Therefore, the apparent binding affinity of AT11-L0 and the AT11-L0/ligands complex towards NCL was also determined by fluorescence titrations using pre-folded AT11-L0 G4 labeled with 5′-Cy5 (Figure 4). 

The titration of the pre-folded AT11-L0 G4 with NCL was fitted with the two-site binding model, indicating that the G4 aptamer interacted with NCL in two different sites. However, the affinity of the aptamer was high for both interaction sites (*K*_D1_ = 0.012 × 10^−9^ M and *K*_D2_ = 91.2 × 10^−9^ M). When complexed with C_8_ or PhenDC3, these affinity constants did not change considerably for both interaction sites (*K*_D1_ = 0.024 × 10^−9^ M and *K*_D2_ = 91.3 × 10^−9^ M for C_8_ and *K*_D1_ = 0.02 × 10^−9^ M and *K*_D2_ = 83.5 × 10^−9^ M for PhenDC3). However, when the AT11-L0 G4 aptamer was complexed with dexamethasone, it appeared to inhibit the interaction between AT11-L0 G4 and NCL. In this case, the more suitable model to obtain the *K*_D_ was the Hill saturation binding model, which indicated that dexamethasone could completely inhibit one of the interaction sites. The *K*_D_ value for the remaining interaction was 0.186 M. This result is in agreement with the data obtained by Noriaki et al., which showed that dexamethasone can interact with NCL and also stimulate its phosphorylation [38].

**Figure 5:**
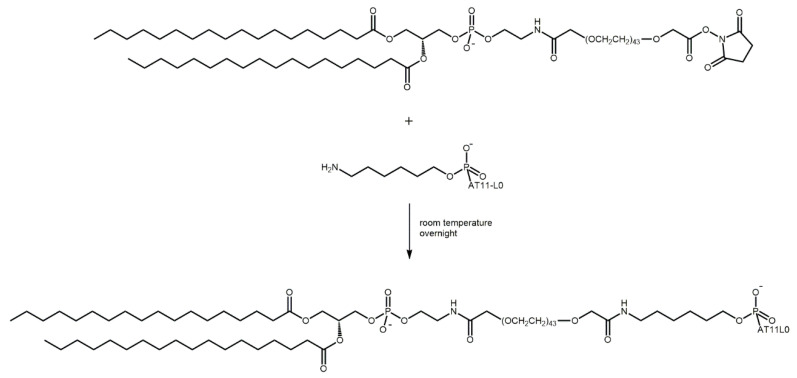
Reactional scheme of the conjugation of NH_2_-AT11-L0 to DSPE-PEG-NHS used to obtain aptamer-functionalized liposomes.

Our results showed that despite being the therapeutic drug used in this study, dexamethasone does not stabilize the G4 in AT11-L0, nor does it promote the interaction between the aptamer and NCL. Thus, to enhance the targeting of tumor cells, the AT11-L0 aptamer was conjugated to liposomes carrying C_8_ or dexamethasone. Figure 5 illustrates the basic design and formulation of 5′-NH_2_-AT11-L0/liposome conjugates. This comprised the assembly of liposomes composed of DSPG, cholesterol, and DSPE-PEG-NHS, followed by conjugation of the aptamer AT11-L0 onto the external surface of liposomes.

The ratio of each lipid used was based on previous work by Xing et al., where the combination of HSPC and DSPE increased the liposome transition temperature and promoted higher rigidity and lower permeability of the formed liposomes [39]. Cholesterol acts as a hydrophobic anchor that increases hydrophobic–hydrophobic interactions in the liposome’s bilayer, as well as liposome rigidity and stability [40]. Finally, PEG modification reduces the nonspecific uptake and promotes higher biocompatibility of the liposomes’ formulations [41]. 

NMR spectra were acquired to evaluate the formation of aptamer-functionalized liposomes (Figure S5) and to assess if the G4 structure was maintained in AT11-L0. The formation of a G-tetrad gives rise to characteristic guanine imino protons, which exhibit their chemical shifts within the range of 10–12 ppm [42] as compared to 13–14 ppm for those involved in Watson–Crick base pairing [43]. Guanine imino protons in a G4 structure also exchange more slowly with solvent than the counterparts in a Watson–Crick duplex [44].

We acquired ^1^H NMR spectra of free AT11-L0 annealed in 65 mM KCl, free liposome, liposome + AT11-L0, and liposome + AT11-L0 + C_8_. These spectra are presented in Figure S5 of the Supplementary Material. 

By comparing these spectra with and without AT11-L0 G4, we concluded the formation of aptamer-functionalized liposomes by observing the imino protons at 10–12 ppm (Figure S3C,D), consistent with the formation of a G4 by an AT11-L0 sequence with its twelve guanines taking part of the G-tetrad core (also see Figure 1C and Figure S1). In addition, we acquired the ^1^H NMR spectrum of liposome + AT11-L0 + C_8_ in 100% D_2_O, and after 6 h the imino protons of guanines in the center G-tetrad exchanged with deuterium of the solvent and disappeared in proton NMR spectra (Figure S5E). The imino protons of guanines in the center G-tetrad exchanged slowly with the solvent and remained detected long after dissolving the sample in D_2_O solution.

In addition, we acquired the UV spectrum of aptamer-functionalized liposomes showing absorbances at 206, 260, and 496 nm of lipid, DNA, and C_8_, respectively (Figure S6), and infrared spectroscopy of liposome + AT11-L0 + C_8_ also showing the guanine C=O stretch mode near 1700 cm^−1^ and Hoogsteen base pairs of the G-tetrad between 1400 and 1600 cm^−1^ (Figure S7). 

The size and the morphology of the formulated liposomes were assessed by scanning electron microscopy (SEM) images and dynamic light scattering (DLS). SEM is useful in understanding whether liposomes are formed and in acquiring information about their morphology [45]. However, the manipulation of the samples before their analysis may lead to the limitation of the obtained images being poorly representative [46]. SEM images (Figure S8) showed that liposomes presented a high homogeneity in terms of size. However, liposome morphology was not uniform and varied from round-shaped liposomes to rod-shaped liposomes, and it is also possible to observe that a small fraction of liposomes formed agglomerates. This result may have been caused by the drying step in the sample preparation, which can induce some morphological changes and agglomeration [47].

DLS was used to evaluate the size and surface zeta potential of the non-functionalized and AT11-L0 aptamer-functionalized liposomes, and the results are shown in Table S2.

The size of empty liposomes without AT11-L0 functionalization was 135.4 nm, with a polydispersity index of 0.141. Regarding drug encapsulation, the size was maintained in the case of C_8_ but not in the case of dexamethasone, where an increase in liposome size was observed. The polydispersity index followed the same tendency. So, the polydispersity index was approximately the same in the case of C_8_, but it was increased in the case of dexamethasone. The functionalization with the AT11-L0 aptamer led to a slight increase in the size of all liposomes. In terms of zeta potential, all liposomes had negative zeta potentials ranging from −22.6 to −36.4 mV. The encapsulation of the drugs led in both cases to less negatively charged liposomes. However, the functionalization with the aptamer did not follow a linear tendency. In the case of empty liposomes and C_8_-loaded liposomes, the aptamer promoted a slight increase of the zeta potential, but in the case of dexamethasone-loaded liposomes, it decreased the zeta potential. These results show that the liposomes that have the aptamer are in the recommended size range, and their charge can increase repulsion between themselves, reducing their aggregation tendency [48,49].

We are interested in liposome-based drug formulations since they present advantages in comparison to free drugs, such as the ability to enhance the delivery of the drugs to the cells of interest and reduce the toxicity of drugs [50]. 

AT11-L0 was efficiently internalized into NCL-positive HeLa cells as shown by our previous report [34]. Following this assumption, we evaluated AT11-L0 liposome C_8_ versus liposome-C_8_ internalization in cancer (A549) and healthy cells (NHDF) by fluorescence confocal microscopy to determine the role played by the aptamer AT11-L0 in cell targeting via NCL and internalization when formulated. The A549 cell line was used since it overexpresses NCL in its cell surface [50]. By contrast, NHDF cells do not overexpress NCL [33], so they were used as a control of low NCL surface expression. For these experiments, we followed the intrinsic fluorescence of C_8_, which was loaded in two different liposomes, while the primary anti-NCL antibody conjugated with the secondary antibody allowed the localization of NCL at the cell surface. The confocal images represented in Figures S9 and S10 and the graph depicted in Figure S11 show that liposomes C_8_ and AT11-L0 liposomes C_8_ were significantly more internalized by cancer cells but without significance when the aptamer was present. Additionally, the colocalization of the liposomes and NCL in the two cell lines was determined by measuring the Manders’ coefficients M1 and M2 (Table S3) [51]. The results showed that the average fraction of liposome C_8_ or AT11-L0 liposome C_8_ colocalized with NCL (M2 coefficient presented in Table S3) was significantly higher in cancer cells when compared with healthy cells.

After the encapsulation of C_8_ and dexamethasone within AT11-L0 aptamer-functionalized liposomes, their capacity of inhibiting the angiogenic process, but without causing toxicity or reducing the cell viability, was evaluated in a HUVEC cell model. In the first approach, the cytotoxicity of free AT11-L0, C_8_, and dexamethasone was evaluated in a HUVEC cell model by using the MTT assay. After that, AT11-L0 aptamer-functionalized liposomes encapsulating C_8_ or dexamethasone were also evaluated using the same method, but only at concentrations of drugs at which cell viability was maintained.

**Figure 6:**
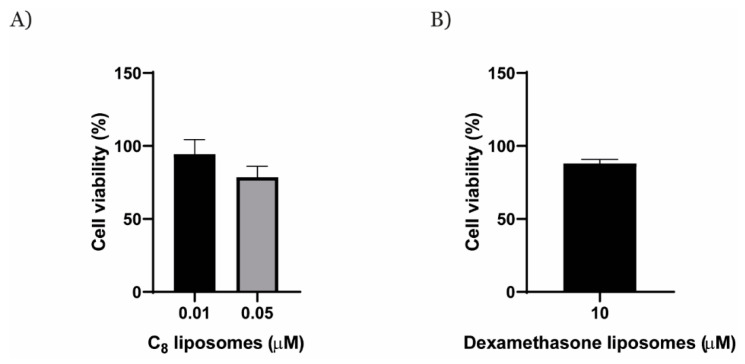
HUVEC cell viability assay in the presence of (**A**) C_8_-loaded liposomes and (**B**) dexamethasone-loaded liposomes.

The obtained data are presented in Figure S12 and Figure 6. Free C_8_ reduced HUVEC viability at concentrations from 0.5 to 5 μM. These results are in agreement with those reported by Carvalho et al., who showed that the viability of normal human dermal fibroblast cells exposed to 1 μM was near 40% [34]. On the other hand, dexamethasone showed a lower effect on HUVEC viability, since higher concentrations (75 to 250 μM) were required to reduce the viability to 50%. AT11-L0 also promoted a considerable reduction in HUVEC viability for concentrations ranging between 2.5 and 20 μM. This result was the most unexpected, since Iturriaga-Goyon et al. reported that AS1411 did not cause a reduction of HUVEC viability in concentrations from 0.75 to 10 μM [29]. However, AS1411 has at least eight polymorphisms, and it is not known which one is the more relevant. So, this result may indicate that AT11-L0 has higher biological activity resulting from having just one predominant G4 topology.

Based on these results, viability tests of AT11-L0 aptamer-functionalized liposomes encapsulating C_8_ or dexamethasone in HUVEC cells should be performed at concentrations of AT11-L0 lower than 2.5 μM. However, the volume of liposomes used is usually defined by the encapsulated drug instead of the AT11-L0 concentration. We followed this approach because the total concentration of AT11-L0 in the liposomes was about 500 nM, which does not have an impact on the cell viability and takes advantage of its targeting ability [34]. Since the main objective of this work was to inhibit the formation of new blood vessels without reducing cell viability, we evaluated the viability of HUVEC cells exposed to C_8_- (0.01 and 0.05 μM) or to dexamethasone-loaded liposomes (10 μM) at concentrations at which the free drugs did not promote an evident decrease in the cell viability in HUVEC cells as well as in other types of healthy and cancer cells [51]. The concentration of dexamethasone-loaded liposome tested was also a result of the maximum concentration that we could achieve through the synthesis process. The results are presented in Figure 6. 

Previously, the cytotoxic effect of C_8_-loaded nanoparticles (nanoaggregates and gold nanoparticles) functionalized with an NCL aptamer (AS1411) was studied [51,52], in which an improvement of the selectivity was found over the free drug. However, C_8_ concentrations were higher (0.5 and 1 µM) than those used in this paper, since the aims of the works were different, and a significant reduction in the viability was desired. Thus, the results from the AT11-L0-functionalized liposomes cannot be compared with those obtained in those reports [52,53].

**Figure 7:**
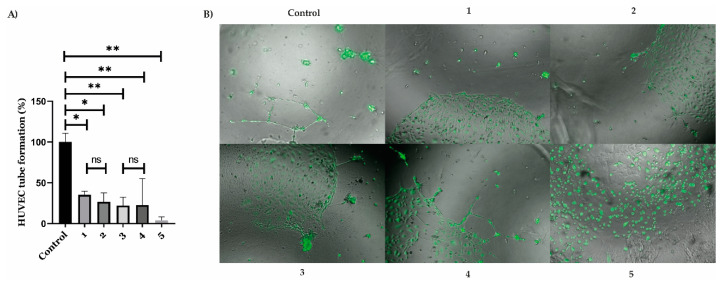
HUVEC tube formation assay. (**A**) Tube formation was significantly inhibited by (1) dexamethasone (10 µM), (2) AT11-L0 dexamethasone-loaded liposomes (10 µM), (3) C_8_ (0.05 µM), (4) AT11-L0 C_8_-loaded liposomes (0.05 µM), and (5) suramin (10 µM) (positive control) in comparison to the control group. * *p* < 0.05 and ** *p* < 0.01. (**B**) Representative images of tube formation assay.

The MTT assay revealed that both AT11-L0 aptamer-functionalized liposomes encapsulating C_8_ and those encapsulating dexamethasone did not considerably affect the viability of HUVEC cells. Therefore, the liposome formulations in the tested concentrations were considered suitable for further evaluation in angiogenesis assays without disturbing normal cell proliferation. Using these concentrations, we tested whether AT11-L0 aptamer-functionalized liposomes encapsulating C_8_ or dexamethasone can inhibit the angiogenic process. The results are presented in Figure 7 in comparison with assays performed with free AT11-L0, C_8_, and dexamethasone.

The data presented in Figure 7 showed that C_8_ has antiangiogenic properties similar to those presented by dexamethasone. It was already known that C_8_ had anticarcinogenic and antiproliferative properties [53]. However, antiangiogenic assays were never performed with this molecule. Since dexamethasone is already used in the treatment of retinal vascular diseases because of its antiangiogenic properties, C_8_ may be a potential candidate to be used in the treatment of these pathologies because it presents similar effects as dexamethasone but at lower concentrations (0.05 µM). Another important advantage of C_8_ is that it can be used in combination with G4 aptamer, since it induces a thermal stabilization of the G4 structure. Nevertheless, AT11-L0 liposomes did not promote a reduction of angiogenesis when compared with the compounds alone. This result can be explained because the concentration of AT11-L0 on the liposomes was about 500 nM. Since AS1411 presented a significant reduction of the angiogenesis at 10 μM, the concentration used in this work may have been too low [29]. However, liposomes conjugated with AT11-L0 can have an important role by increasing the delivery of C_8_ and dexamethasone in cells that overexpress NCL. In the angiogenic assay, HUVEC cells were VEGF-dependent, which means they have an overexpression of VEGF (and consequently of cell surface NCL). In retinal eye disease, only some cells have this overexpression, and therefore liposomes conjugated with AT11-L0 may be useful to help the compounds reach only cells with overexpression of VEGF and NCL that will trigger the angiogenesis process.

## 3. Discussion

AT11-L0-functionalized liposomes were designed to improve the antiangiogenic effects of C_8_ and dexamethasone by reduction of tubes in the HUVECs model. AT11-L0 formed a majority of the parallel-stranded G4 structure with a well-defined profile at 65 mM KCl and also bound NCL. C_8_ strongly interacted with AT11-L0 and stabilized the structure by more than 6 °C, leading to the formation of a G4 complex distinct from the free form. Dexamethasone presented the opposite results towards AT11-L0. The binding affinity between AT11-L0 and NCL in the presence of dexamethasone showed that the ligand affected the recognition of NCL by AT11-L0 G4. For this reason, dexamethasone was encapsulated on the liposome as a therapeutic molecule instead of a G4 ligand. In the case of C_8_, it did not impair recognition by NCL. A cell viability study suggested no cytotoxic effect on HUVEC cells after treatment with AT11-L0 aptamer-functionalized liposomes encapsulating C_8_ or dexamethasone. The ligands alone demonstrated a more pronounced cytotoxic effect on HUVEC cells.

In addition, AT11-L0 aptamer-functionalized liposomes encapsulating C_8_ or dexamethasone did not present a significant reduction in the angiogenic process when compared to the free-tested ligands (dexamethasone and C_8_). 

## 4. Materials and Methods

### 4.1. Reagents

The AT11-L0 sequence (5′-TGGTGGTGGTTGTTGGGTGGTGGTGGT-3′) and the ds26 (5′-CAATCGGATCGAATTCGATCCGATTG-3′) sequence were purchased from Eurogentec (Belgium) with HPLC-grade purification and a purity of 98%. In addition, the labeled sequences 5′-Cy5-AT11-L0, 5′-FAM-AT11-L0-TAMRA-3′, and 5′-NH_2_-AT11-L0-3′ were purchased from the same company.

Lyophilized oligonucleotides were resuspended in Milli-Q water without any additional purification step and stored at −20 °C until its usage.

The absorbance of each stock solution of oligonucleotide was measured at 260 nm to determine the oligonucleotide concentration. The measurements were performed on a UV–Vis spectrophotometer (Thermo Scientific™ Evolution 220, Saint Louis, MO, USA). The molar extinction coefficients used for the calculations were provided by the manufacturer and presented in Table 2. Before each experiment, the oligonucleotide was annealed in 20 mM potassium phosphate buffer (pH 6.9) containing 65 mM KCl (annealing buffer) by heating for 10 min at 95 °C followed by an ice cooldown of 10 min.

Ligands PhenDC3 (3,3′-[1,10-phenanthroline-2,9-diylbis(carbonylimino)]bis[1-methylquinolinium] 1,1,1-trifluoromethanesulfonate; CAS: 929895-45-4;), TMPyP4 (tetra-(N-methyl-4-pyridyl)porphyrin; CAS: 36951-72-1), BRACO-19 (N,N′-(9-(4-(dimethylamino)phenylamino)acridine-3,6-diyl)bis(3-(pyrrolidin-1-yl)propanamide); CAS: 1177798-88-7) and PDS 4-(2-aminoethoxy)-N2,N6-bis[4-(2-aminoethoxy)-2-quinolinyl]-2,6-pyridinedicarboxamide; CAS: 1085412–37–8) were acquired from Sigma Aldrich (St. Louis, MO, USA) and stocked on a 10 mM DMSO solution. Synthesis and purification of the ligand C_8_ (10-(8-(4-iodobenzamide)octyl))−3,6-bis(dimethylamine) acridinium iodide was performed as previously described [54]. Dexamethasone (CAS: 50-02-2) was acquired from Tokyo chemical industry (Tokyo, Japan), and a 10 mM DMSO stock solution was prepared. The recombinant NCL RBD1,2 used in all experiments was synthesized and purified as reported previously [53,54].

Cholesterol (CAS: 57-88-5), 1,2-distearoyl-sn-glycero-3-phospho-(1′-rac-glycerol) (sodium salt) (DSPG) (CAS: 200880-42-8) and 1,2-distearoyl-sn-glycero-3-phosphoethanolamine-N-[carboxy(polyethylene glycol)-2000, NHS ester] (sodium salt) (DSPE-PEG) (CAS: 2410279-87-5) were acquired from Avanti Polar Lipids (Birmingham, AL, USA).

### 4.2. Circular Dichroism

AT11-L0 was dissolved in Milli-Q water at a concentration of 10 µM and annealed as described before. Experiments were performed using a 1 mm path-length quartz cell (Hellma, Germany) on a Jasco J-815 spectropolarimeter (Tokyo, Japan) equipped with a Peltier temperature controller (model CDF-426S/15).

CD spectra were performed setting the spectral width to 220–340 nm, with a scan speed of 200 nm/min, 1 nm bandwidth, and 1 s integration time over 3 averaged accumulations. To start, AT11-L0 was titrated with increasing concentrations of KCl (1 to 65 mM) by adding the required volume of KCl from a 1 M stock directly into the quartz cell.

For ligand titrations, the same protocol was employed. AT11-L0 was annealed with 65 mM of KCl prepared in 20 mM of phosphate buffer (pH 6.9). For each ligand titration point, the required volume of ligand was added to the cell containing the pre-folded AT11-L0 G4.

CD-melting experiments were performed to obtain the melting temperature (*T*_m_) for each ligand titration point (0, 0.5, 1.0, and 2.0 molar equivalents). These experiments were performed with a heating rate of 2 °C/min by monitoring the maximum ellipticity at 260 nm in a temperature range from 20 to 100 °C.

The results were converted into fraction folded plots (θ), through Equation (1), which were adjusted to a Boltzmann distribution, using OriginPro2021.

(1) $$\theta =\frac{CD-{CD}_{260\;nm}^{min}}{{CD}_{260\;nm}^{max}-{CD}_{260\;nm}^{min}}$$

The melting temperature (*T*_m_) was calculated through the two-state mid-transition point. The CD value is the molar ellipticity at 260 nm at each temperature and CD^min^ and CD^max^ are the lowest and highest molar ellipticities, respectively.

### 4.3. NMR Spectroscopy

Unlabeled AT11-L0 sequence was dissolved in Milli-Q water (pH 6.9) containing 10% (*v*/*v*) D_2_O (Eurisotop, France) at a concentration of 100 µM in a 5 mm tube with a total volume of 600 µL and titrated with increasing concentrations of KCl (1 to 65 mM) and annealed at each point as described above. Liposome samples were mixed with 10% D_2_O/90% H_2_O to perform 180 μL on a 3 mm NMR tube. The NMR spectra were acquired using a 600 MHz Bruker Avance III equipped with a 5 mm inverse detection z gradient QCI cryoprobe operating at a Larmor ^1^H frequency of 600.10 MHz at 293.15 K. The water suppression pulse sequence used was zgesgp (according to the Bruker standard library), with a 2 s relaxation delay, 32 K data points, 256 scans, and a 20.019 Hz spectral width centered on the water resonance. Chemical shifts (δ) were measured in ppm. Bruker TopSpin4.1 was used to analyze all the spectra obtained.

To assuring that AT11-L0 was bound to liposomes, NMR spectra were acquired of free AT11-L0, free liposome, and conjugated with the AT11-L0 and loaded with C_8_. The spectra for both samples were compared to evaluate if the AT11-L0 is bound to liposome and if the G4 structure was formed.

### 4.4. Förster Resonance Energy Transfer (FRET)-Melting Experiments

For FRET-melting experiments, FAM-AT11-L0-TAMRA was used at a concentration of 0.2 μM, and the ligand concentrations used were 0.2 μM (1 molar equivalent), 0.4 μM (2 molar equivalents), 0.6 μM (3 molar equivalents), and 1 μM (5 molar equivalents). The oligonucleotide was annealed as described before in 20 mM potassium buffer (pH 6.9) supplemented with 65 mM KCl. FRET-melting competition studies were performed with 3 μM (15 molar equivalents) or 10 μM (50 molar equivalents) of ds26 (a double-stranded sequence) in the presence of FAM-AT11-L0-TAMRA at 0.2 μM and the ligands at 0.6 μM (3 molar equivalents). Thereafter, the samples were incubated for 30 min at room temperature. After 30 min incubation, samples were incubated on a thermocycler (CFX Connect™ Real-Time PCR (RT-PCR) Detection System (Bio-Rad, Hercules, CA, USA), supplied with a FAM filter (λ_ex_ = 492 nm; λ_em_ = 516 nm) at 25 °C during 5 min followed by temperature increases of 1 °C at each 1 min, from 25 °C until 95 °C. FAM emission was measured each time the temperature was increased. Each condition was tested in duplicate on three independent 96-well RT-PCR plates. Data curves were normalized to calculate the melting temperatures. To assess the competition effect, the Selectivity factor (S factor) was determined following Equation (2).

(2) $$S\;\mathrm{factor}=\frac{\Delta T_{m}\mathrm{in}\;\mathrm{presence}\;\mathrm{of}\;\mathrm{the}\;\mathrm{competitor}}{\Delta T_{m}\mathrm{without}\;\mathrm{the}\;\mathrm{competitor}}$$

### 4.5. Fluorescence Spectroscopy

Fluorescence titrations were carried out on a Horiba FluoroMax4 fluorometer (Tokyo, Japan) equipped with a Peltier-type temperature control system, using a quartz suprasil cuvette (10 × 4 mm) filled with a volume of 700 µL.

Each spectrum obtained results from an acquisition over three averaged scans using an emission slit width of 2 nm, an excitation slit width of 1 nm, and an integration time of 0.5 s. The wavelength used for the excitation of Cy5-AT11-L0 was 647 nm. Titrations were performed by consecutively adding to the previously annealed Cy5-AT11-L0 (1 µM) the required volume of ligand or NCL directly from stock solutions. Each addition was followed by 10 min of equilibrium.

Fluorescence data obtained were converted into a fraction of bound ligand (α) plots using Equation (3):

(3) $$\alpha =\frac{I-I_{\lambda }^{\mathrm{free}}}{I_{\lambda }^{\mathrm{bound}}-I_{\lambda }^{\mathrm{free}}}$$

where I is the fluorescence intensity at 667 nm for each DNA:ligand ratio, and I^free^ and I^bound^ are the fluorescence intensities of the free and fully bound DNA, respectively. Data points were then fitted into the Hill saturation binding model or the two-site bind model using OriginPro 2021, and *K*_D_ values were determined from Equation (4) for the Hill saturation binding model and Equation for the two-site bind model (5):

(4) $$\alpha =\frac{{\left[\mathrm{DNA}\right]}^{n}}{K_{D}+{\left[\mathrm{DNA}\right]}^{n}}$$

(5) $$\alpha =\frac{\left[\mathrm{DNA}\right]}{K_{D1}+[\mathrm{DNA}]}+\frac{\left[\mathrm{DNA}\right]}{K_{D2}+[\mathrm{DNA}]}$$

in which *K*_D_, *K*_D1_, and *K*_D2_ are the apparent equilibrium dissociation constants, [DNA] is the concentration of the DNA, and n is the Hill constant which defines the cooperativity of ligand binding.

### 4.6. Synthesis of Liposomes Functionalized with AT11-L0 and Ligands

Synthesis of liposomes was based on the thin film hydration method followed by sequential extrusion, and AT11-L0 was conjugated on the surface of liposomes as previously described with some modifications. Briefly, Cholesterol, DSPG, and DSPE-PEG (in a molar ratio of 2:1:0.16 of DSPG:Cholesterol:DSPE-PEG) [39,55] solubilized in chloroform were mixed in a 2 mL Eppendorf that was left open overnight for solvent evaporation. Afterward, the lipid thin film formed was weighted to calculate the total lipid mass. Then, the lipid film layer was hydrated with PBS to recover a lipids solution with 1 mg/mL. Additionally, lipid film layers were also hydrated with PBS containing C_8_ (for a final mass ratio of 0.025:1 of C_8_:lipids) or dexamethasone (for a final mass ratio of 0.1:1 of dexamethasone:lipids). After that, the solution was heated at 65 °C and then quickly frozen and thawed (with liquid nitrogen and on a heat block at 40 °C) for 5 cycles. The size of liposomes was homogenized by multiple extrusion steps (11 times) through polycarbonate membranes with a pore size of 0.1 µm using a mini-extruder set from Avanti Polar Lipids, Inc (Birmingham, AL, USA). Then, the resulting liposomes were washed with milli-Q water by ultrafiltration using an ultrafiltration device with a cut-off of 2 kDa (Sartorius, Germany) to remove the small molecular weight residues, four times at 200 RCF for 40 min.

To prepare the AT11-L0 conjugated liposomes, NH_2_-AT11-L0 was incubated with liposomes (in a mass ratio of 1:10 of DNA:liposome), during an overnight with gentle agitation. The unreacted NH_2_-AT11-L0 was removed by four rounds of centrifugation at 200 RCF for 40 min. After that, samples were stocked at 4 °C until used.

### 4.7. Structural Characterization of Liposomes

Liposomes were characterized by dynamic light scattering (DLS) to obtain information about the size and the polydispersity of liposomes. First, 1 mL of the liposome’s solution at the concentration obtained by the synthesis process was directly inserted into a disposable 1 cm beam plastic cell using a Zetasizer Nano ZS equipment (Malvern Instruments, Malvern, UK) and the Malvern Zetasizer software v6.34. Each sample was measured 3 times at 25 °C. SEM was used to confirm the morphology and the aggregation state of liposomes. To perform SEM, 20 μL of the sample at the concentration obtained in the synthesis process was put on a circular coverslip at room temperature and let dry overnight protected from direct sunlight. The coverslips containing the dried samples were fixed in a metallic sample holder with two-sided tape. The sample holder was inserted into the anode stage of an Emitech K550 (London, UK) sputter coater. The coater was vacuum closed, and the samples were gold coated for 1.5 min. Samples were observed using a Hitachi S-2700 (Tokyo, Japan) scanning electron microscope, with an accelerating voltage of 20 kV using various magnifications until reached a good image of liposomes.

### 4.8. Confocal Fluorescence Microscopy

A549 and NHDF cell lines were grown in Ham’s F12 medium and RPMI, respectively, both containing 10% (*v*/*v*) FBS and 1% (*v*/*v*) penicillin–streptomycin. The cells were seeded at a density of 5 × 10^4^ cells per well (200 μL in each well) in a treated μ-slide 8 well (ibidi, Germany) and grown at 37 °C in a 5% CO_2_ humidified atmosphere during an overnight.

Cells were incubated with C_8_-loaded liposomes or AT11-Lipsomes loaded with C_8_ for 2 h. Then, the cells were incubated with the primary anti-NCL polyclonal antibody (PA3-16875, Invitrogen, Waltham, MA, USA) at 1:100 for 2 h at 37 °C, and, subsequently, with a secondary antibody (Alexa Fluor 647^®^, A21244, Invitrogen, USA; dilution of 1:1000) for 1 h at 37 °C. Additionally, the nucleus was stained by incubation with 1 μM of Hoechst 33342^®^ (h3570, Invitrogen™ Thermo Fisher Scientific, Waltham, MA, USA) for 15 min. Between each step, the sample was washed off by rinsing with PBS three times. Cells were sighted using a Zeiss LSM 710 confocal laser scanning microscope (Zeiss, Germany) coupled with a plane-apochromat with a 40×/DIC M27 objective to capture the fluorescence images. Fluorescence intensity was obtained through the Zen 2.3 blue Software.

### 4.9. MTT Assay

The MTT assay was used to evaluate the cellular viability when exposed to liposome formulations. For this assay, Human Umbilical Vein Endothelial Cells (HUVEC) (ATCC, PCS-100-010, Manassas, VA, USA) were grown on vascular basal cell medium (ATCC, PCS-100-030, Manassas, VA, USA) supplemented with an Endothelial Cell Growth Kit-VEGF (ATCC, PCS-100-041, Manassas, VA, USA) and harvested when reaching 80–90% confluency. Then seeded on 96 wells using 100 μL of the same medium with the referred supplementation at a density of 5 × 10^3^ cells per well. After 24 h, the medium was removed, and it was added new medium containing the compounds or the formulations of liposomes that we wanted to evaluate at distinct concentrations (0.005 to 5 μM of C_8_, 5 to 250 μM of dexamethasone, 0.25 to 20 μM of AT11-L0, 0.01 and 0.05 μM of C_8_-loaded liposomes and 10 μM of dexamethasone-loaded liposomes). After 72 h, the medium was removed, and 100 μL of a 3-[4,5-dimethylthiazol-2-yl]-2,5 diphenyl tetrazolium bromide (MTT) solution was added at a concentration of 1 mg/mL. After 4 h, MTT was removed, and the insoluble formazan crystals were completely dissolved on DMSO. Finally, absorbance was read on a plate reader (Bio-rad xMark spectrophotometer from Bio-rad, Hercules, CA, USA) at 570 nm.

### 4.10. Angiogenesis Assay

The anti-angiogenic properties of the tested compounds/liposomes were tested using an angiogenesis assay kit (In vitro)(Abcam, Cambridge, UK), according to the manufacturer’s guidelines. Briefly, HUVEC were harvested when reaching 80–90% confluency and seeded on 96-well plates using 50 μL of extracellular matrix (Matrigel) provided with the kit at a density of 5 × 10^3^ cells per well. Negative controls were seeded without this matrix. The compounds/liposomes were added at the desired concentrations (10 μM of dexamethasone and dexamethasone-loaded liposomes, and 0.05 μM of C_8_ and C_8_-loaded liposomes), and suramin was added to another negative control. Cells were incubated with the compounds/formulations for 18 h. Then they were incubated with the staining dye provided with the kit, for 30 min, after being carefully washed and finally observed on a microscope using light and fluorescence. Results were analyzed using ImageJ software, and tube formation was calculated based on the total length of segments formed by HUVEC.

## Figures and Tables

**Table 1:** Selectivity factor of BRACO-19, C_8_, PDS, PhenDC3, and TMPyP4 for AT11-L0 G4 in the presence of 15 and 50 molar equivalents of ds26.

	S Factor	
Ligand	15 Molar eq. ds26	50 Molar eq. ds26
BRACO-19	0.66	0.45
C_8_	0.47	0.38
PDS	0.83	0.73
PhenDC3	0.94	0.89
TMPyP4	0.51	0.33

**Table 2:** Molar extinction coefficient of oligonucleotides.

Name	Molar Extinction Coefficient (L·mol^−1^·cm^−1^)
AT11-L0	256.1
FAM-AT11-L0-TAMRA	317.1
Cy5-AT11-L0	266.1
NH_2_-AT11-L0	256.1
ds26	253.2

## Data Availability

Data is contained within the article and supplementary material.

## Supplementary Materials

The following supporting information can be downloaded at: https://www.mdpi.com/article/10.3390/ph16050751/s1, Table S1: *T*_m_ of AT11-L0 with C_8_, dexamethasone, and PhenDC3 at different concentrations determined by CD melting; Figure S1: 1H NMR spectra of unlabeled AT11-L0 obtained with increasing concentrations of KCl in 20 mM of phosphate buffer; Figure S2: CD melting spectra of AT11-L0 in the absence and presence of increased molar equivalents of ligands (A) PhenDC3, (B) dexamethasone, and (C) C_8_. CD spectra were acquired in a buffer containing 20 mM of potassium buffer (pH 6.9) and 65 mM of KCl; Figure S3: CD spectra and melting of AT11-L0 in 20 mM KPi + 65 mM KCl of unlabelled (in red) and dye-labeled (in black) AT11-L0; Figure S4: Fluorescence titration spectra of pre-folded 5′-Cy5-AT11-L0 G4 with increasing concentrations of (A) C_8_, (B) PhenDC3, and (C) dexamethasone. The experiments were performed with 65 mM of KCl in 20 mM potassium buffer with excitation set at 647 nm, and emission was recorded ranging from 660 to 800 nm; Figure S5: 1H NMR spectra of (A) free AT11-L0 annealed in 65 mM KCl; (B) free liposome; (C) liposome +AT11-L0; (D) liposome +AT11-L0 + C8; (E) liposome +AT11-L0 + C_8_ after 6h in 100% D2O; Figure S6: UV spectrum of liposome +AT11-L0 + C_8_; Figure S7: FTIR spectra of (A) liposome and (B) liposome+AT11-L0+C_8_; Figure S8: SEM image of liposomes functionalized with AT11-L0; Table S2: Size, zeta potential, and polydispersity index of the tested liposomes; Figure S9: Fluorescence confocal microscopy images of A549 (cancer cells) and NHDF (healthy cells) cells incubated with liposome-C8 for 2 h. Cell nuclei are stained with Hoechst 33342 in the blue channel, nucleolin is dyed with AlexaFluor 647^®^ (red) and C8 emits green fluorescence. Scale bar is 50 µm; Figure S10: Fluorescence confocal microscopy images of A549 (cancer cells) and NHDF (healthy cells) incubated with AT11-L0 liposome C8 for 2 h. Cell nuclei are stained with Hoechst 33342 in the blue channel, nucleolin is dyed with AlexaFluor 647^®^ (red) and C_8_ emits green fluorescence. Scale bar is 50 µm; Figure S11: Mean fluorescence intensity per cell obtained by fluorescence confocal microscopy of C8-loaded liposomes or AT11-L0 liposomes C_8_ in A549 and NHDF cells after 2 h incubation. Values are presented as mean values ± standard deviation (SD). *** *p* = 0.003 compared to A549 cells treated with liposomes C_8_; #### *p* < 0.0001 relatively to A549 cells treated with AT11-L0—liposomes with C_8_; Table S3: Colocalization coefficients of NCL and liposome C_8_ or AT11-L0 liposome C_8_ represented in mean ± SD; Figure S12: HUVEC cell viability assay in the presence of A) C_8_, B) dexamethasone and C) AT11-L0;
