# Supplementary material for: Assessment of Aptamer as a Potential Drug Targeted Delivery for Retinal Angiogenesis Inhibition
Source: Pharmaceuticals (Basel). 2023 May 16;16(5):751. doi: 10.3390/ph16050751 (PMC10221175; doi:10.3390/ph16050751)
Supplement: Supplementary file 1 [file pharmaceuticals-16-00751-s001.zip › pharmaceuticals-2259708-supplementary.pdf]

Assessment of aptamer as a potential drug targeted delivery for retinal angiogenesis inhibition

David Moreira[1], Jéssica Lopes-Nunes[1], Fátima Milhano Santos[2], Maria Paula Cabral Campello,[3,4] Maria Cristina Oliveira[3,4], António Paulo,[3,4] Cândida Tomaz[1,5*] and Carla Cruz[1,5*]

[1] CICS-UBI - Health Sciences Research Centre, University of Beira Interior, Covilhã, Portugal,
[2] Functional Proteomics Laboratory, Centro Nacional de Biotecnología, CSIC, Calle Darwin 3, Campus de Cantoblanco, 28049 Madrid, Spain.
[3]Centro de Ciências e Tecnologias Nucleares, Instituto Superior Técnico, Universidade de Lisboa, Estrada Nacional 10 (km 139.7), 2695-066 Bobadela, Portugal
[4] Departamento de Engenharia e Ciências Nucleares, Instituto Superior Técnico, Universidade de Lisboa, Estrada Nacional 10 (km 139.7), 2695-066 Bobadela LRS, Portugal
[5]Departamento de Química, Universidade da Beira Interior, Rua Marquês de Ávila e Bolama, 6201-001 Covilhã, Portugal
[*] Correspondence: carlacruz@fcsaude.ubi.pt; ctomaz@ubi.pt

Table S1 – T_m of AT11-L0 with C_8, dexamethasone, and PhenDC3 at different concentrations determined by CD melting.

ΔTm (°C)			
**Ligand**	**0.5 molar eq.**	**1 molar eq.**	**2 molar eq.**
**PhenDC3**	2.15	2.27	6.67
**C_8**	0.91	2.91	6.68
**Dexamethasone**	0.32	1.03	1.71

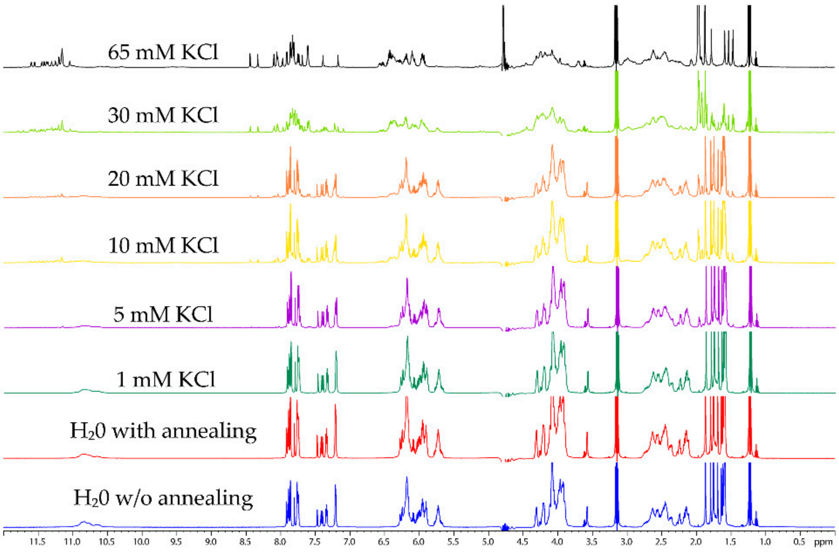

Figure S1- ^{1}H NMR spectra of unlabeled AT11-L0 obtained with increasing concentrations of KCl in 20 mM of phosphate buffer.

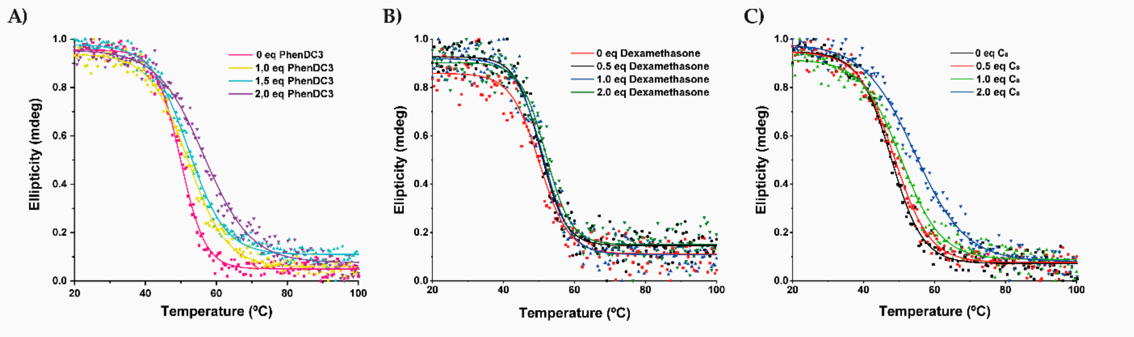

Figure S2- CD melting spectra of AT11-L0 in the absence and presence of increased molar equivalents of ligands (A) PhenDC3, (B) dexamethasone, and (C) C_8. CD spectra were acquired in a buffer containing 20 mM of potassium buffer (pH 6.9) and 65 mM of KCl.

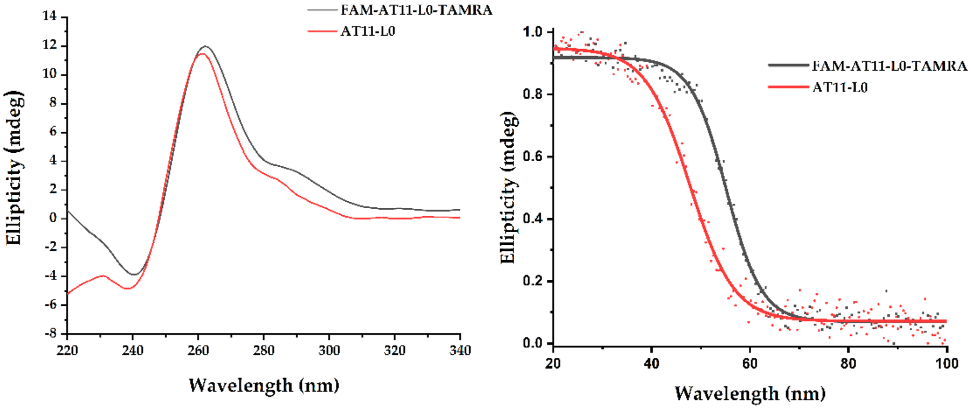

Figure S3- CD spectra and melting of AT11-L0 in 20 mM KPi + 65 mM KCl of unlabelled (in red) and dye-labeled (in black) AT11-L0.

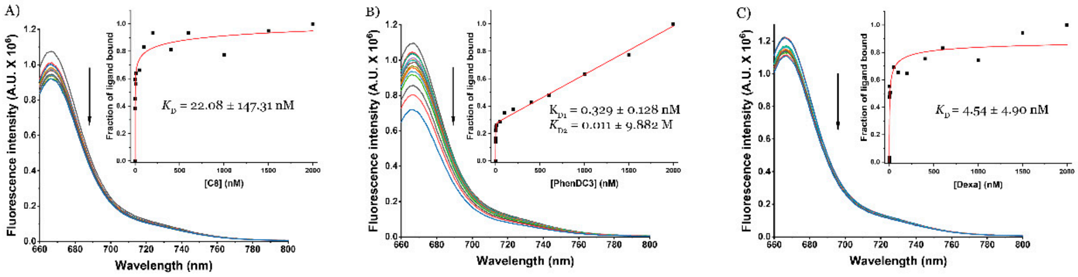

Figure S4 – Fluorescence titration spectra of pre-folded 5'-Cy5-AT11-L0 G4 with increasing concentrations of (A) C_8, (B) PhenDC3, and (C) dexamethasone. The experiments were performed with 65 mM of KCl in 20 mM potassium buffer with excitation set at 647 nm, and emission was recorded ranging from 660 to 800 nm.

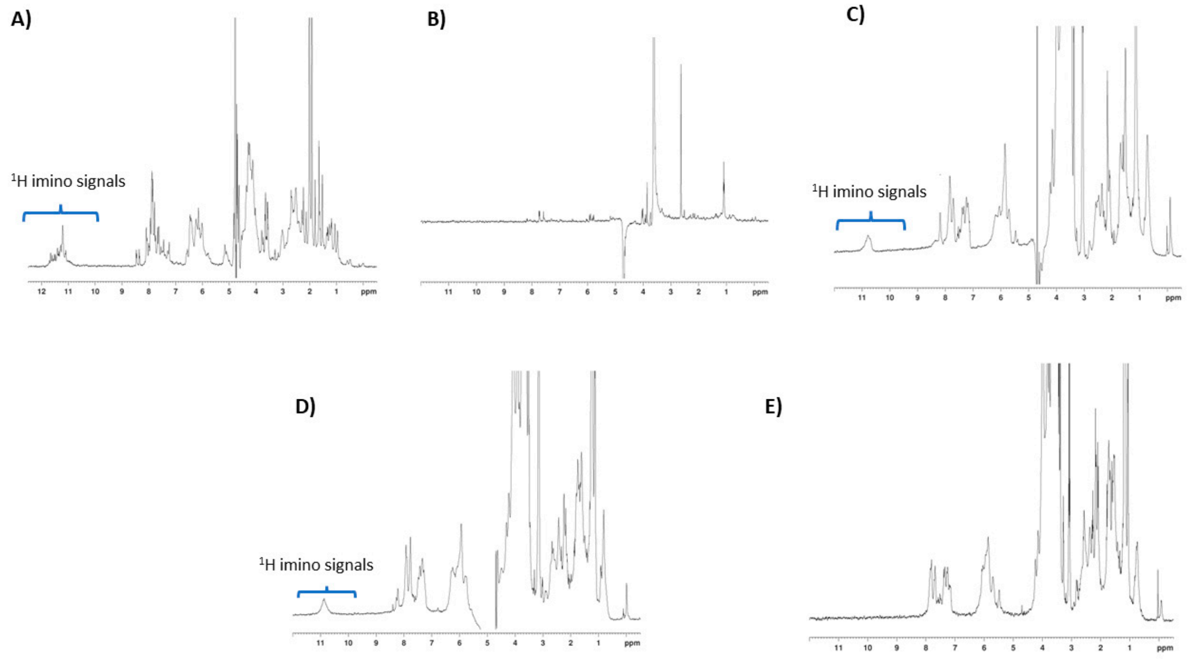

Figure S5 – ^{1}H NMR spectra of **(A)** free AT11-L0 annealed in 65 mM KCl; **(B)** free liposome; **(C)** liposome +AT11-L0; **(D)** liposome +AT11-L0 + C_8; **(E)** liposome +AT11-L0 + C_8 after 6h in 100% D_2O.

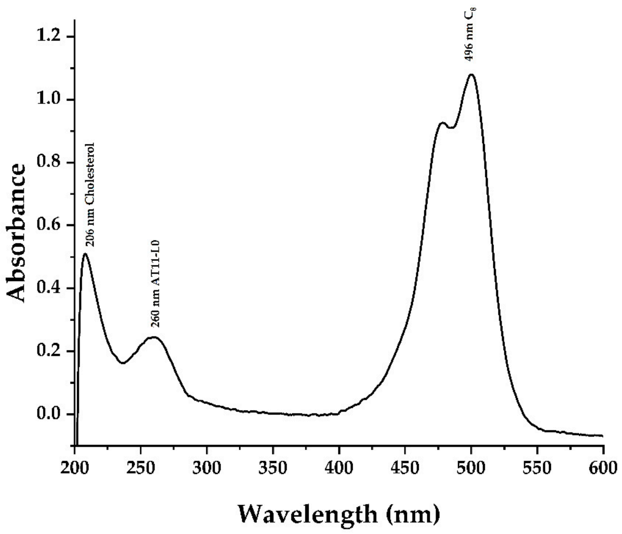

Figure S6– UV spectrum of liposome +AT11-L0 + C_8.

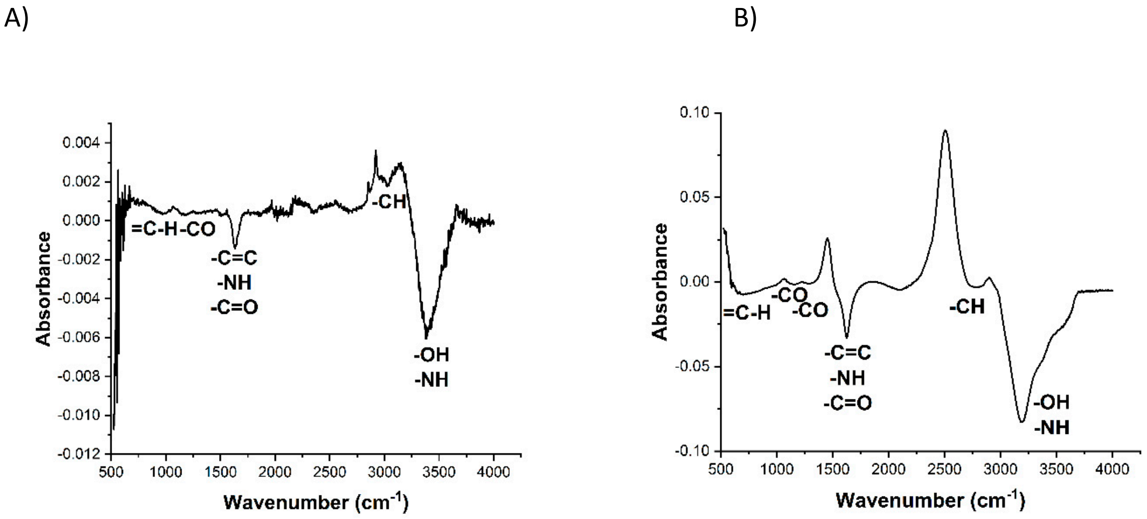

Figure S7- FTIR spectra of (A) liposome and (B) liposome+AT11-L0+C_8.

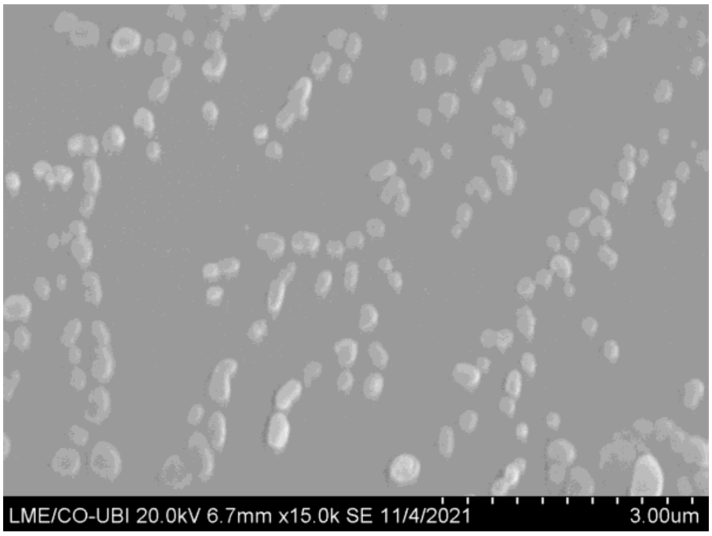

Figure S8 – SEM image of liposomes functionalized with AT11-L0.

Table S2 – Size, zeta potential, and polydispersity index of the tested liposomes

Liposomes	Size (nm)	Polydispersity Index	Zeta potential (mV)
Empty	135.4	0.141	-36.4
Empty and aptamer functionalized	150.0	0.152	-34.6
C_8 loaded	134.1	0.156	-26.2
C_8 loaded and aptamer functionalized	135.4	0.231	-22.6
Dexamethasone loaded	214.4	0.474	-22.6

Dexamethasone loaded and aptamer functionalized	215.8	0.392	-27.8

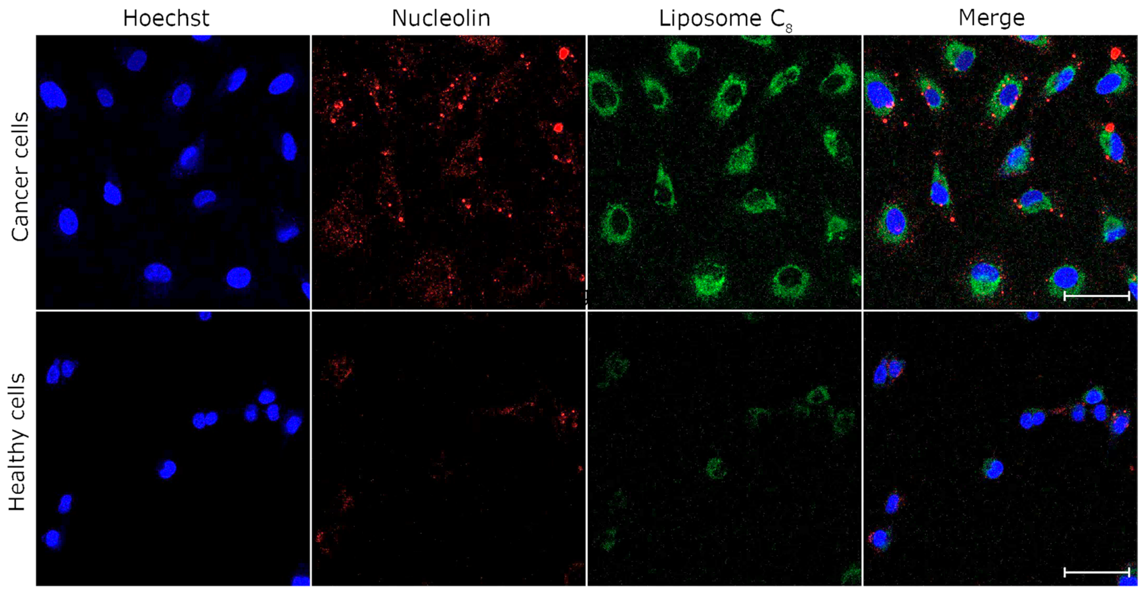

Figure S9- Fluorescence confocal microscopy images of A549 (cancer cells) and NHDF (healthy cells) cells incubated with liposome-C_8 for 2 h. Cell nuclei are stained with Hoechst 33342 in the blue channel, nucleolin is dyed with AlexaFluor 647® (red) and C_8 emits green fluorescence. Scale bar is 50 µm.

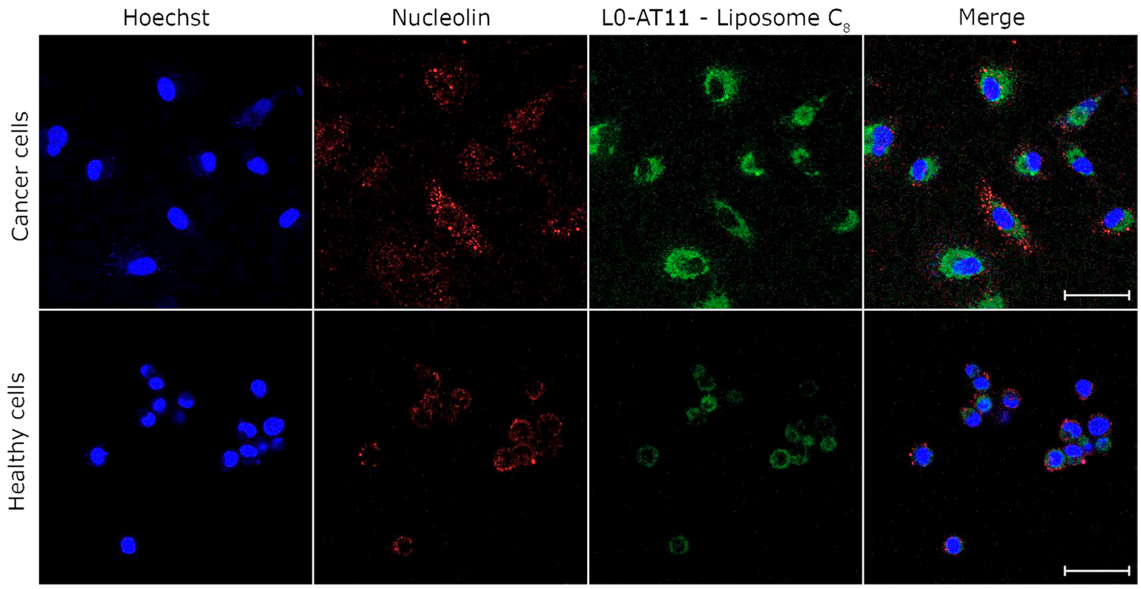

Figure S10- Fluorescence confocal microscopy images of A549 (cancer cells) and NHDF (healthy cells) incubated with AT11-L0 liposome C_8 for 2 h. Cell nuclei are stained with Hoechst 33342 in the blue channel, nucleolin is dyed with AlexaFluor 647® (red) and C_8 emits green fluorescence. Scale bar is 50 µm.

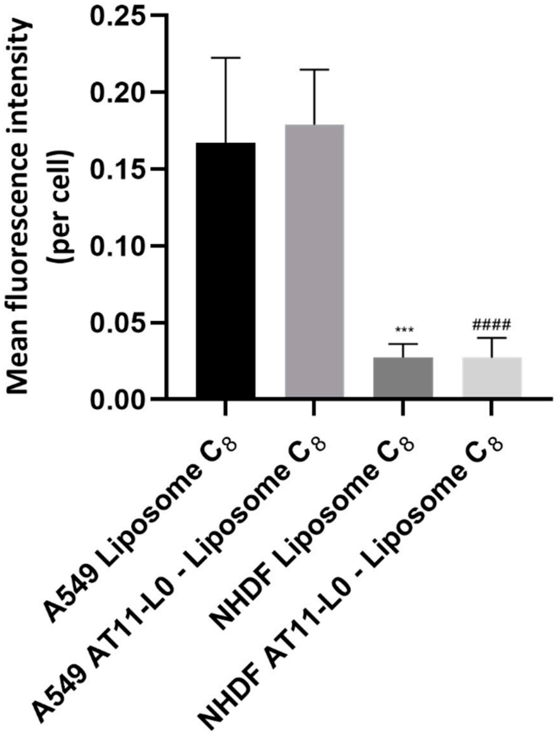

Figure S11- Mean fluorescence intensity *per* cell obtained by fluorescence confocal microscopy of C_8-loaded liposomes or AT11-L0 liposomes C_8 in A549 and NHDF cells after 2 h incubation. Values are presented as mean values ± standard deviation (SD). ***p=0.003 compared to A549 cells treated with liposomes C_8; ####p<0.0001 relatively to A549 cells treated with AT11-L0 - liposomes with C_8.

Table S3- Colocalization coefficients of NCL and liposome C_8 or AT11-L0 liposome C_8 represented in mean ± SD.

		**Manders' Coefficient**	
		M1	M2
**A549 cells**	**liposome C_8**	0.8275 ± 0.1727	0.9588 ± 0.006994
	**AT11-L0 liposome C_8**	0.8918 ± 0.04892	0.9634 ± 0.004615
**NHDF cells**	**liposome C_8**	0.1843 ± 0.07499[a]	0.8535 ± 0.03223[b]
	**AT11-L0 liposome C_8**	0.1765 ± 0.06707[c]	0.7890 ± 0.03642[c; d]

M1 represents fraction of NCL overlapping liposome C_8 or AT11-L0 liposome C_8. M2 represents fraction of liposome loaded C_8 or AT11-L0 liposome C_8 overlapping NCL staining. [a]p<0.0001 and [b]p=0.0001 relatively to A549 cells treated with liposomes C_8. [c]p<0.0001 relatively to A549 cells treated with AT11-L0 liposomes C_8. [d]p=0.0096 relatively to NHDF cells treated with liposomes C_8.

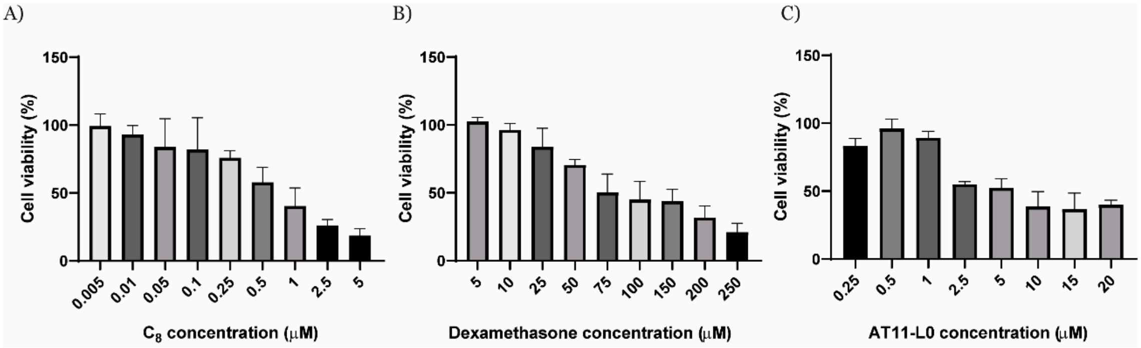

Figure S12– HUVEC cell viability assay in the presence of A) C_8, B) dexamethasone and C) AT11-L0.
